# Urinary angiostatin: a novel biomarker of kidney disease associated with disease severity and progression

**DOI:** 10.1186/s12882-019-1305-2

**Published:** 2019-04-03

**Authors:** Yuan-Yuan Xia, Ru Bu, Guang-Yan Cai, Xue-Guang Zhang, Shu-Wei Duan, Jie Wu, Di Wu, Xiang-Mei Chen

**Affiliations:** Department of Nephrology, Chinese PLA General Hospital, Chinese PLA Institute of Nephrology, State Key Laboratory of Kidney Diseases, National Clinical Research Center for Kidney Diseases, Beijing, 100853 China

**Keywords:** Urinary protein, Angiostatin, IgA nephropathy, Disease severity, Biomarker

## Abstract

**Background:**

This study aimed to evaluate the value of urinary angiostatin levels for assessing disease severity and progression of IgA nephropathy (IgAN).

**Methods:**

Urinary angiostatin was identified as one of the distinct proteins in samples of patients with IgAN analyzed by Raybiotech protein array, and further confirmed by enzyme-linked immunosorbent assay (ELISA).

**Results:**

Urinary angiostatin levels were significantly higher in IgAN patients than that in healthy controls (HC) subjects and lower than in disease controls (DC) patients. The concentrations of angiostatin in urine normalized to urinary creatinine (angiostatin/Cr) were positively associated with proteinuria level. With advancing chronic kidney disease (CKD) stage, urinary angiostatin/Cr levels were gradually increased. Urinary angiostatin/Cr levels in patients with Lee’s grade IV–V were significantly higher than those in Lee’s grade I–II and III. We further compared urinary angiostatin/Cr levels by using Oxford classification and found the expression in patients with mesangial proliferative score 1(M1) was significantly higher than that in M0 (*P* < 0.001). In addition, the levels of urinary angiostatin/Cr in patients with tubular atrophy/interstitial fibrosis score 1(T1) and T2 were significantly higher than those in T0 (*P* < 0.01, *P* < 0.001, respectively). After follow-up, renal survival was significantly worse in patients with higher levels of urinary angiostatin (*P* < 0.05).

**Conclusions:**

Urinary angiostatin may be a useful novel noninvasive biomarker to evaluate disease severity and progression of IgAN.

**Electronic supplementary material:**

The online version of this article (10.1186/s12882-019-1305-2) contains supplementary material, which is available to authorized users.

## Background

IgA nephropathy (IgAN) is the most common form of glomerulonephritis worldwide, and accounts for approximately 45% of primary glomerular disease in the Chinese population [1, 2]. Many studies have showed that 25% of patients with IgAN progressed to end-stage renal disease (ESRD) within 10–20 years of the onset of the disease [3]. Renal pathology such as the Oxford histological classification and Lee’s grade classification is the best approach to assess disease severity and provide prognostic value beyond that explained by clinical parameters [4, 5]. However, risk of bleeding and other clinical complications limits the application of biopsy. In addition, sometimes number of glomerulus acquired by renal biopsy is not enough to accurately assess the kidney lesion of IgAN. Therefore, it is necessary to develop noninvasive and sensitive biomarkers for evaluating disease severity in patients with IgAN. Because it can be obtained noninvasively in large quantities, urine has become one of the most attractive biofluids with which to find noninvasive biomarkers for kidney diseases. Over the past few years, urine proteomics have extensively been used for the study of novel noninvasive biomarkers of IgAN. However, only few novel biomarkers have yet been identified and the same marker was expressed differently at different stages of IgAN, which reflected the complexity of screening for IgAN biomarkers [6–8]. Screening non-invasive biomarkers with high sensitivity and specificity for early diagnosis and prognosis of IgAN has become a challenge in clinical practice.

The technique of protein microarray is a rapid and high-output approach that promotes more and more practical and broad applications [9]. We have used protein microarray technology to find biomarkers of kidney disease. In the present study, we screened for urine proteomes that are differentially expressed in patients with biopsy-proven IgAN using protein microarray. We then performed cohort validation by ELISA analysis to identify the urine biomarkers for disease specificity and severity of IgAN. In general, we found the expression of urinary angiostatin levels in IgAN were significantly different in healthy control subjects and disease control subjects. The marker was correlated with clinical parameters and was significantly elevated in advanced chronic kidney disease (CKD) and in advanced pathological changes. Patients with IgAN who have a higher urinary angiostatin levels had an unfavorable prognosis.

## Methods

### Enrollment of patients and control subjects

A total of 229 primary IgAN patients were enrolled in our study. 169 non-IgAN renal disease patients with biopsy-proven evidence were recruited as disease controls, including patients with MN (*n* = 104), MCD (*n* = 40), and FSGS (*n* = 25). All patients attended an inpatient clinic at the Chinese PLA General Hospital from August 2013 through April 2017. Forty-five gender- and age-matched healthy volunteers were also recruited for urine collection as healthy controls. Healthy volunteers were enrolled from the staff of the Chinese PLA General Hospital and had no known kidney or systemic disease.

### Ethics approval and consent to participate

The study protocol was approved by the ethics committee of the Chinese PLA General Hospital (Beijing, China) in accordance with the Declaration of Helsinki (S2014–004-002). The consent obtained from the participants was written.

### Inclusion and exclusion criteria

Inclusion criteria were the following: age ≥ 14 years, eGFR ≥15 (ml/ min per 1.73 m^2^) had received a renal biopsy at the Chinese PLA General Hospital, and had complete medical records on file.

Exclusion criteria were the following: diagnosis of a secondary IgAN, infection, pregnancy, lactation, or systemic disease.

### Study design

**Fig. 1:**
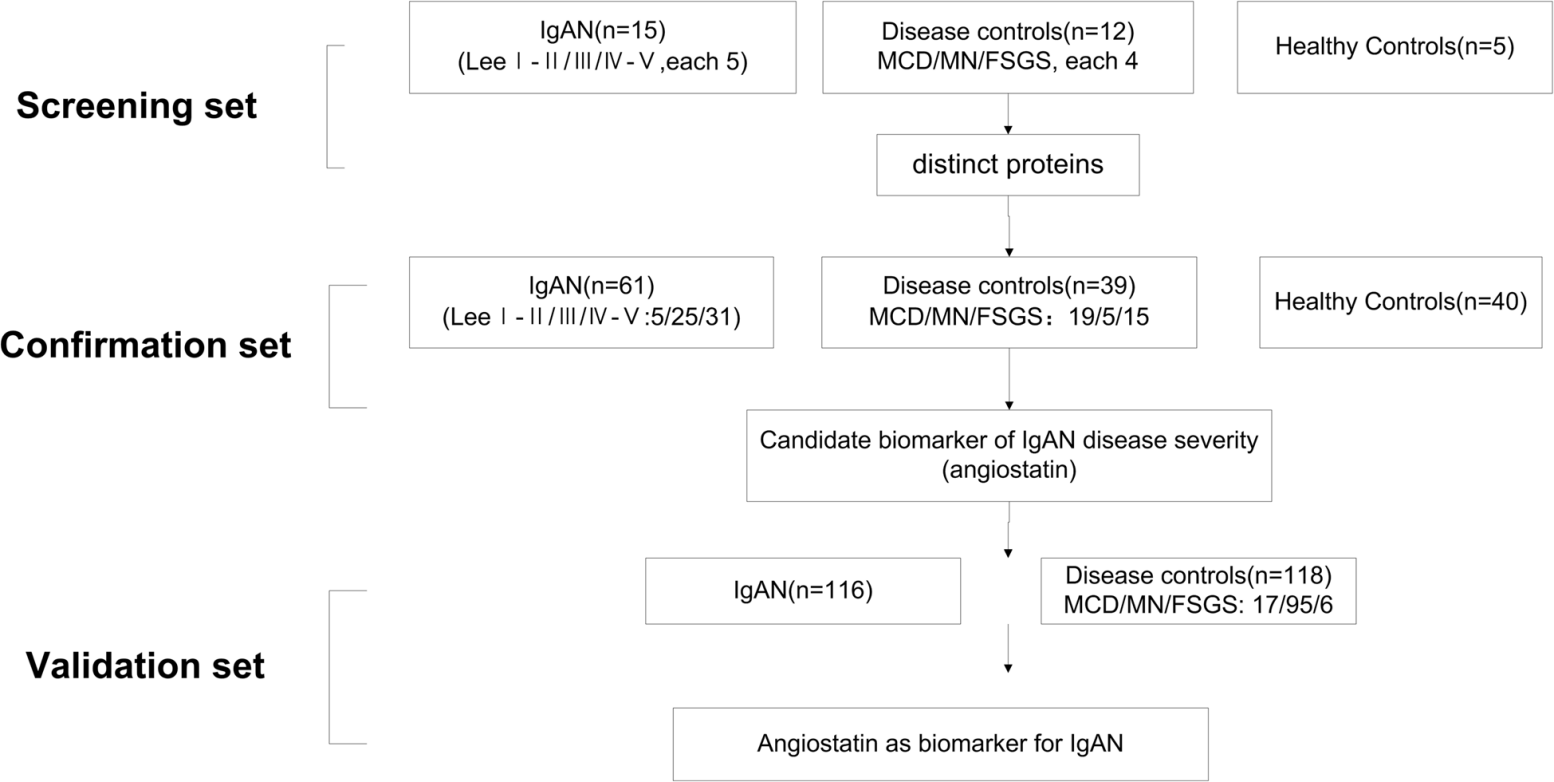
Research design for identifying IgAN disease severity using urinary angiostatin

The research approach of this study is shown in Fig. 1. First, we used a Raybiotech protein array and urine samples from IgAN (*n* = 15; Lee’s gradeI–II/III/ IV–V, 5 in each), DC (*n* = 12; MN/MCD/FSGS, 4 in each), and HC (*n* = 5) to identify differential protein expression (screening stage). The urinary angiostatin levels were significantly higher in IgAN with Lee’s grade IV–V than in healthy controls, and were then confirmed by ELISA methods using urine samples from a larger cohort of 61 patients with IgAN, 39 disease controls, and 40 healthy controls (confirmation stage). Next, we measured urinary angiostatin concentrations in another cohort of 116 IgAN patients and 118 non-IgAN patients. We combined all 177 patients with IgAN to determine whether urinary angiostatin levels were a biomarker of disease severity in IgAN (validation stage). In addition, patients with IgAN were followed up, and 87 patients met the requirement with a median follow-up time of 17 (14–30) months. We also measured serum angiostatin concentrations in another cohort of 31 IgAN patients.

### Urine and serum collection and Raybiotech protein array analysis and angiostatin detection

Fresh samples of midstream urine before biopsy were collected into a clean specimen cup and centrifuged at 4 °C for 30 min (3000 g/min) to remove urine sediment. Serum from patients within biopsy less than 6 months were collected into a clean specimen cup and centrifuged at 4 °C for 10 min (3000 r/min). Urine supernatant and serum were aliquoted prior to storage at − 80 °C until the next analysis. Urinary samples were treated with RayBio® AAH-CYT-G4000–8 kit and screening criteria of difference between groups is ratio > 1.5 or < 0.67. Urinary and serum angiostatin was quantified by a standard sandwich ELISA assay using a human angiostatin ELISA kit (RayBio® Human Angiostatin ELISA Kit).

### Clinical characteristics and some definitions

For each enrolled patient with IgAN, the following parameters at the time of renal biopsy were collected: sex, age, blood pressure, 24-h urine protein excretion, serum creatinine, 24-h urinary creatinine concentration, cystatin C and Lee’s grades, and Oxford classification. The demographics of patients in the IgAN cohort and the DC group are summarized in Table 1. The estimated glomerular filtration rates (eGFR) were calculated using the CKD Epidemiology Collaboration (CKD-EPI) equation, and the patients were categorized into CKD stages 1–5 according to the Kidney Disease Outcomes Quality Initiative (K-DOQI) practice guideline.

**Table 1:** The demographics and clinical characteristics of patients in the IgAN group and in the disease control group

	IgAN	DC	*P* value
Sex (male/female)	106/71	91/66	0.704
Age (years)	35.62 ± 11.87	41.81 ± 13.70	< 0.001
MAP (mmHg)	100.02 ± 12.60	97.40 ± 12.33	0.055
TP (g/L)	64.24 ± 8.99	51.29 ± 10.74	< 0.001
Alb (g/L)	38.06 ± 6.37	27.80 ± 8.2	< 0.001
Scr (μmol/L)	100.10 (77.08, 148.95)	72.50 (58.85, 93.710)	< 0.001
UA (μmol/L)	398.58 ± 103.35	360.61 ± 105.06	0.001
BUN (mmol/L)	6.91 ± 3.87	6.17 ± 4.44	0.103
Proteinuria (g/24 h)	1.41 (0.72, 2.95)	3.18 (1.65, 5.21)	< 0.001
< 1 *n* (%)	65 (36.7)	24 (15.3)	
1~3.5 *n* (%)	80 (45.2)	58 (36.9)	
≥ 3.5 *n* (%)	32 (18.1)	75 (47.8)	
CKD stage	177	157	< 0.001
CKD1 stage *n* (%)	56 (31.6)	110 (70.1)	
CKD2 stage *n* (%)	51 (28.8)	27 (17.2)	
CKD3 stage *n* (%)	54 (30.5)	15 (9.6)	
CKD4 stage *n* (%)	16 (9.1)	5 (3.2)	

At each timepoint, the following parameters were recorded in the IgAN patients: blood pressure, proteinuria, and serum creatinine. The composite outcomes were defined as a 50% decrease in eGFR or reaching ESRD.

The original renal biopsies were reviewed by two pathologists and scored using the Oxford Classification of IgAN (MEST score). The MEST score consisted of mesangial hypercellularity (M0 ≤ 0.5; M1>0.5), endocapillary hypercellularity (E0 absent; E1 present), segmental glomerulosclerosis (S0 absent; S1 present), and tubular atrophy/interstitial fibrosis (T0 ≤ 25%; T1 25–50%; T2 ≥ 50%).

### Statistical analysis

Statistical analyses were performed by SPSS software 19.0. The quantitative variables were expressed as mean ± standard deviation (SD) or median and interquartile range (IQR), as appropriate. Differences of quantitative parameters between groups were assessed using the one-way analysis of variance (ANOVA),the Kruskal–Wallis test, or independent sample t test. As the urinary angiostatin/Cr levels coincide with log normal distribution, so we performed logarithmic conversion of urinary angiostatin/Cr levels and then compared differences between groups using the one-way analysis of variance (ANOVA) or in dependent sample t test. In order to avoid the difference of eGFR level and proteinuria between IgAN and DC group, CKD stage and 24 h proteinuria group were used as covariates, and the propensity score matching was matched by 1: 1, and accuracy was set to 0.2. The urinary angiostatin levels between groups and different CKD stages were compared in matched subjects. The Spearman’s coefficient correlation was applied to analyze the correlation between urinary angiostatin/Cr levels and clinical parameters. For the analysis of factors correlated with urinary angiostatin/Cr, we further explored using multivariable linear regression analysis. Kaplan–Meier curves were used to analyze the renal survival with the use of a log-rank test. A *p*-value of < 0.05 was considered to be statistically significant.

## Results

### Identification of angiostatin in IgA nephropathy

**Fig. 2:**
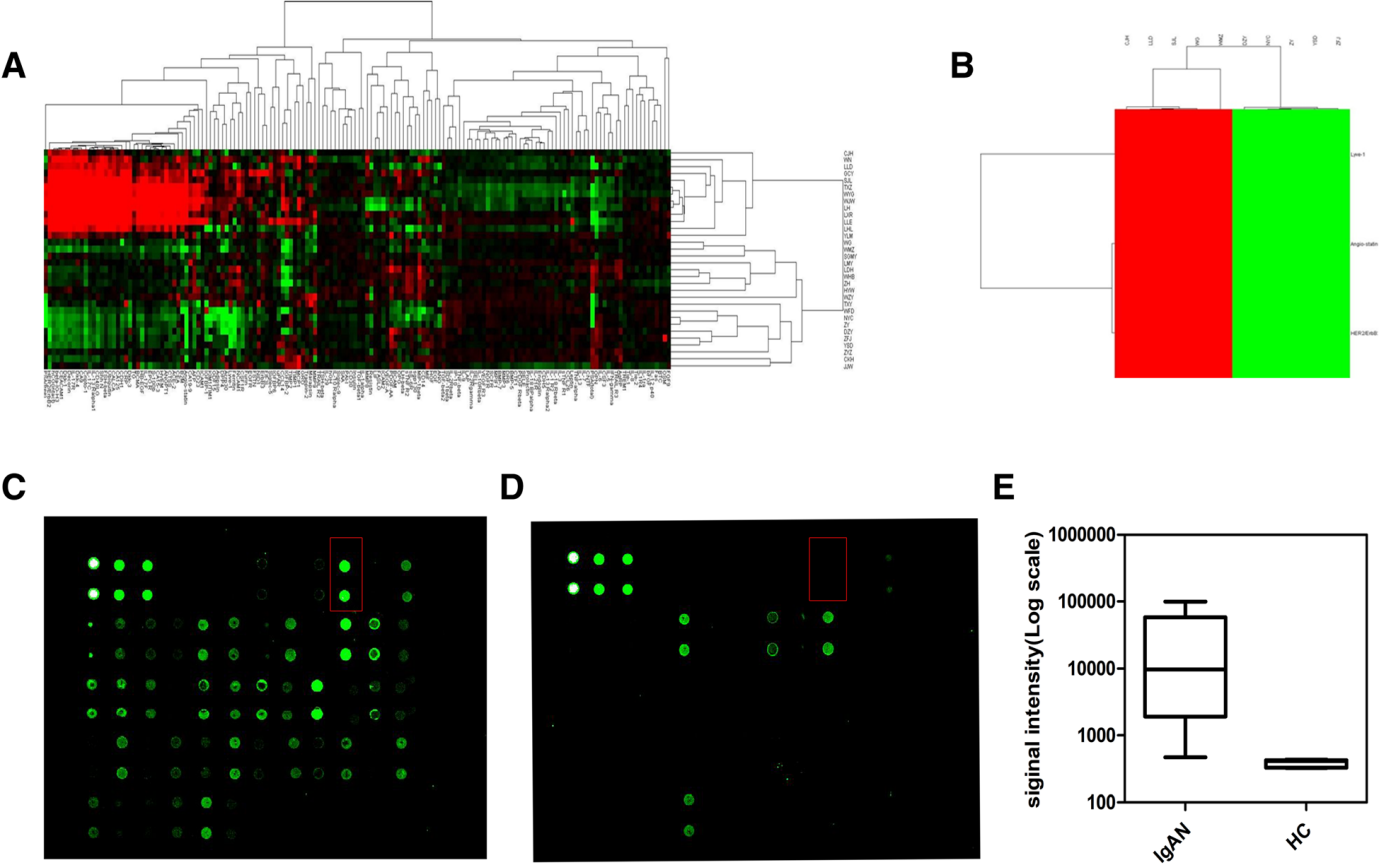
Screening of differential expression of urinary proteins in IgAN patients. Heat map of urinary differential protein expression profiles in (**a**) IgAN patients, disease control patients, and healthy control subjects, and (**b**-**d**) IgAN patient’s with Lee’s IV-V and healthy control subjects based on the Raybiotech protein array analysis of the urine samples. **e** Expression of angiostatin was increased in IgAN patients compared with healthy controls (*p* = 0.0079)

The Raybiotech protein array identified distinct proteins among 15 IgAN patients (groups by Lee’s grade I-II, III, and IV-V. 5 in each group), 12 non-IgAN glomerular disease control (DC) patients (minimal change nephropathy (MCD), membranous nephropathy (MN), and focal segmental glomerulosclerosis (FSGS), 4 in each group), and 5 healthy volunteers. The clinical information of the IgAN patients was shown in Additional file 1: Table S1. The microarray analysis showed that a total of 51 proteins with altered levels in the urine of patients with IgAN were selected compared with other two groups (Fig. 2a). Among the protein molecules screened whose differential expression ratio > 1.5, the urinary angiostatin levels were significantly higher in IgAN patients with Lee’s IV–V than in healthy controls (Fig. 2b-e, *P* = 0.0079). We then performed an ELISA analysis to confirm the urinary levels of some distinct proteins in a confirmation set and found that urinary angiostatin levels were significantly higher in the IgAN group than in the HC group (*P*<0.001; Fig. 2a). The clinical information obtained from the healthy subjects was shown in Additional file 1: Table S2.

### Evaluation of angiostatin for disease specificity and severity of IgAN

We further obtained samples from more patients with IgAN and analyzed data by combining the confirmation set. The clinical information of patients with IgAN and DC groups was shown in Table 1. Lee’s grade and Oxford Classification scores characteristics of patients in the IgAN group was shown in Additional file 1: Table S3.

**Fig. 3:**
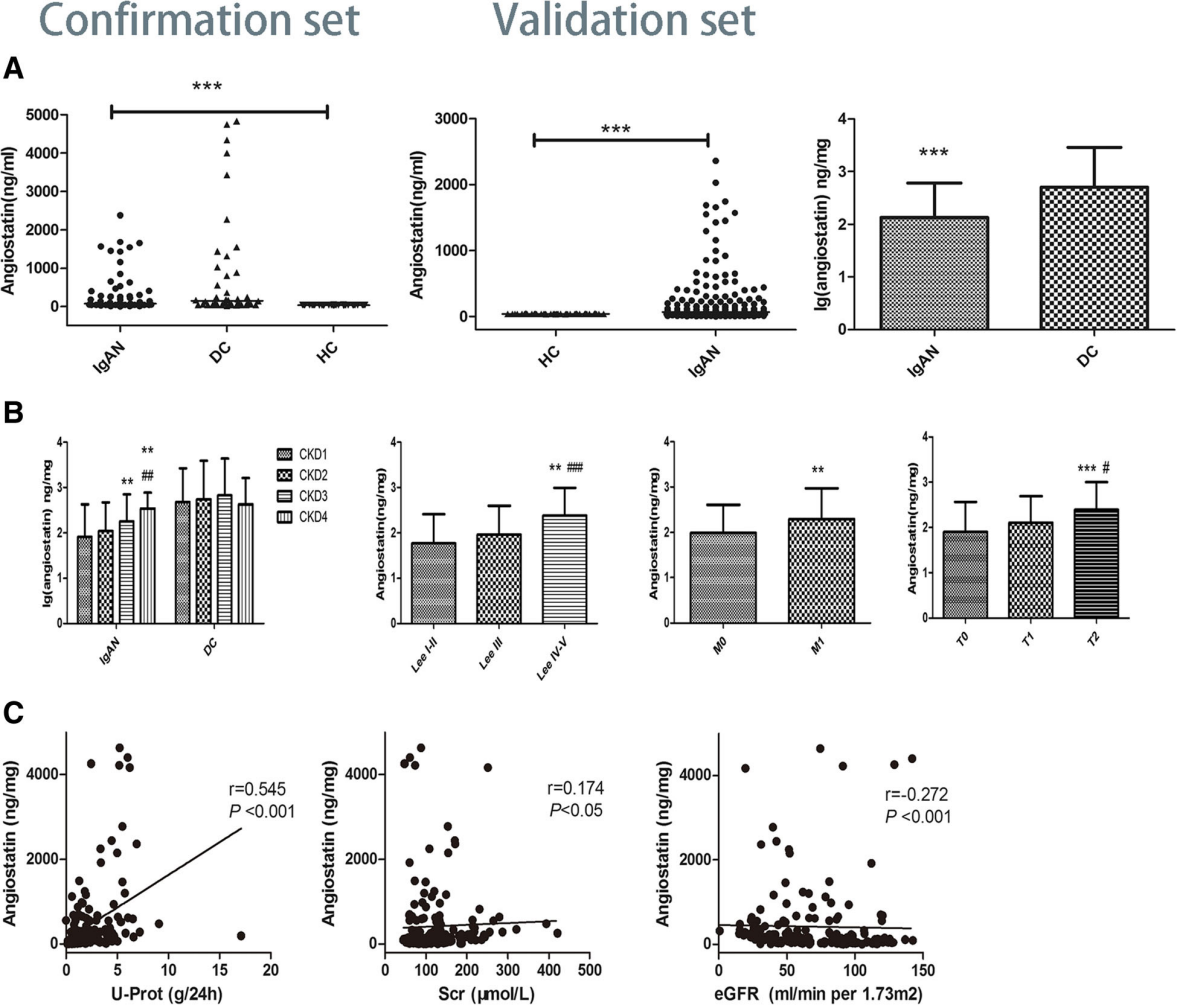
Levels of urinary angiostatin reflect disease specificity and severity. **a**. Levels of urinary angiostatin in the confirmation set and validation set. Levels of urinary angiostatin/Cr in the validation set. (When compared with healthy controls and disease controls: ***P and ###*P* < 0.001). **b**. Levels of urinary angiostatin/Cr in IgAN patients with different CKD stages, Lee’s grades, mesangial proliferative and tubular atrophy, and interstitial fibrosis change scores (compared with CKD stage 1, Lee’s grade IV-V, M0, and T0, respectively; **P < 0.01 and ***P < 0.001). **c**. Spearman correlation analysis of urinary angiostatin/Cr levels with proteinuria levels, serum creatinine, eGFR in IgAN patients

Clearly, urinary angiostatin levels were significantly higher in all IgAN patients (72.66(38.33–226.32) ng/mL) than in HC subjects (38.57(37.85–39.84) ng/mL, *P* < 0.001) and lower than in DC patients (358.59 (96.09–1196.59) ng/mL, *P* < 0.001, Fig. 3a). The concentration of angiostatin in urine normalized to urinary creatinine (angiostatin/Cr: 113.38(51.00,327.64) ng/mg, lg (angiostatin/Cr): 2.13 ± 0.66) were significantly lower in IgAN patients than in DC patients (620.61(126.00,1644.93) ng/mg, lg (angiostatin/Cr): 2.70 ± 0.75P < 0.001, Fig. 3a).

Compared with CKD stage1, the levels of urinary angiostatin were significantly higher in CKD stage≥3(CKD stage3: 182.68(70.38, 466.12) versus (vs) CKD stage1: 82.15(34.80, 147.28), *P* < 0.01; CKD stage4: 275.81(211.08,521.24) vs CKD stage1: 82.15(34.80, 147.28), *P* < 0.001)). The patients in CKD stage 2(72.15(38.15, 327.32), lg (angiostatin/Cr): 2.05 ± 0.65) had the same levels compared to the patients in CKD stage1 (Fig. 3b). No similar results were found in patients in the disease control group.

After 1:1 propensity score matching, 85 patients with IgAN and DC patients were analyzed, the clinical information obtained from the IgAN patients was shown in Additional file 1: Table S4. There was no difference in eGFR, CKD stage and proteinuria level between the groups. Urinary angiostatin/Cr levels in IgAN were lower than those in DC groups((193.62(90.47,1075.82) vs 785.78(236.99,2191.86), lg (angiostatin/Cr):2.45 ± 0.70 vs 2.89 ± 0.67, *P* < 0.001) after matched. But no difference was found in different CKD stages in IgAN, which may be caused by few patients in CKD stage 4 and 5 after matched.

Compared to patients with Lee’s pathological grade IV–V (220.14 (93.25,561.78) ng/mg, lg (angiostatin/Cr):2.38 ± 0.61), the levels of urinary angiostatin/Cr were decreased significantly in Lee’s pathological grade III (73.68 (39.14,196.28) ng/mg, lg (angiostatin/Cr):2.00 ± 0.63, *P* < 0.001) and grades I–II (44.11 (29.07,247.53) ng/mg, lg (angiostatin/Cr):1.77 ± 0.64, *P* < 0.01; Fig. 3b). We further compared urinary angiostatin/Cr levels by different Oxford Classification scores. The levels of urinary angiostatin/Cr in patients with mesangial proliferative score 1(M1: 192.30(71.82, 545.25) ng/mg, lg (angiostatin/Cr):2.29 ± 0.68) were significantly higher than that in M0 (88.16(42.00, 223.37) ng/mg, lg (angiostatin/Cr):2.00 ± 0.62, *P* < 0.01; Fig. 3b). The levels of urinary angiostatin/Cr in patients with tubular atrophy/interstitial fibrosis score 0(T0: 66.75 (34.17,211.46) ng/mg, lg (angiostatin/Cr):1.90 ± 0.66) were significantly lower than that in T1(103.75 (63.64,198.59) ng/mg, lg (angiostatin/Cr):2.11 ± 0.58, *P* < 0.01) and T2 (247.53 (103.75,589.02) ng/mg, lg (angiostatin/Cr):2.41 ± 0.66, *P* < 0.001; Fig. 3b). However, no difference was found in endocapillary hypercellularity or segmental glomerulosclerosis lesions in IgAN patients. We compared serum angiostatin levels in different Lee’s pathological grades and Oxford Classification scores and no difference was found.

The levels of urinary angiostatin/Cr were positively correlated with the levels of 24-h proteinuria (r = 0.552, *P* < 0.001) and serum creatinine (r = 0.192, *P* < 0.05) in patients with IgAN. Moreover, a negative correlation was found between the levels of urinary angiostatin/Cr and eGFR (r = − 0.284, *P* < 0.001, Fig. 3c). A similar result was found between urinary angiostatin/Cr levels and proteinuria in patients in the disease control group (r = 0.583, *P* < 0.001). However, no correlation was observed between urinary angiostatin/Cr and serum creatinine or between urinary angiostatin/Cr and eGFR in disease control patients.

To identify the independent factors correlated with urinary angiostatin/Cr level, we then applied a multivariable linear regression model to make further analysis. After adjusting for gender, age, MAP, serum creatinine, mesangial proliferation, tubular atrophy/interstitial fibrosis, and Lee’s grade, proteinuria level was independently associated with urinary angiostatin level (Table 2).

**Table 2:** Multivariate analysis of the relation between urinary levels of angiostatin/Cr and clinical-histopathological parameters of patients with IgAN

	β-coefficient	*P*-value	95% CI	
			Lower	Upper
Gender	0.13	0.062	−0.132	0.132
Age	0.034	0.664	− 0.119	0.186
MAP (mmHg)	−0.015	0.840	−0.158	0.129
Scr (μmol/L)	−0.583	0.561	−0.269	0.147
Proteinuria (g/24 h)	0.442	< 0.001	0.295	0.589
eGFR (ml/min per 1.73 m^2^)	0.162	0.172	−0.071	0.395
Mesangial proliferation	0.068	0.349	−0.075	0.210
Tubular atrophy/interstitial fibrosis	0.126	0.196	−0.066	0.319
Lee’s Grades (I-II/III/IV-V)	0.062	0.451	−0.100	0.224

### Renal outcomes and urinary angiostatin level in lgAN

**Fig. 4:**
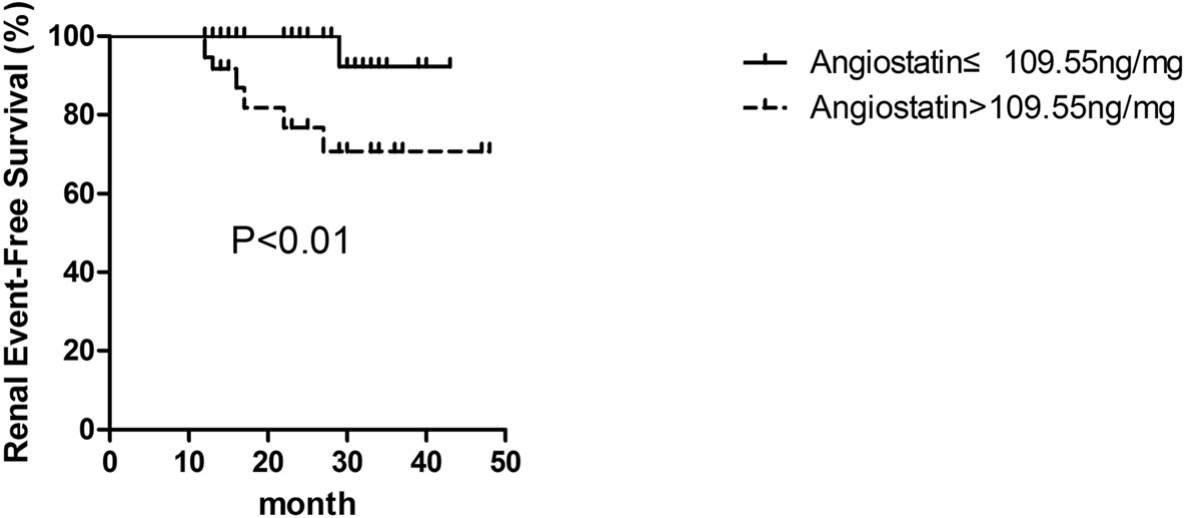
Renal survival of patients with IgAN with lower and higher levels of urinary angiostatin. The green line represents patients with lower levels of urinary angiostatin (< 109.55 ng/mg). The blue line represents patients with higher levels of urinary angiostatin (≥109.55 ng/mg)

To evaluate the prognostic effect of urinary angiostatin on disease progression in IgAN, we divided the second cohort of IgAN patients into two groups according to the median level of urinary angiostatin/Cr (109.55 ng/mg). The median follow-up duration was 17 months (14–30 months). Renal survival was significantly worse in patients with higher urinary angiostatin level (*P* < 0.01; Fig. 4).

## Discussion

In this study, a total of 51 differential proteins were recognized from the urine samples of patients with IgAN by protein microarray screening. Urinary angiostatin level was higher in patients with IgAN than in healthy control subjects, which were selected for further analysis in a large cohort.

This is the first study to investigate whether urinary angiostatin could be a reliable noninvasive biomarker for monitoring renal injury and predicting disease progression in patients with IgAN. We found urinary angiostatin levels were higher in IgAN patients than in healthy controls and lower than in disease controls. The marker was elevated significantly in advanced CKD patients, whereas no difference was found between CKD stages 1 and 2. We further compared the levels of urinary angiostatin in different histopathological changes. Urinary angiostatin levels in Lee’s grade IV–V were significantly higher than in Lee’s grade I–II and III. Urinary angiostatin levels were markedly changed with different severity of mesangial proliferation and tubular atrophy/interstitial fibrosis. The higher levels of urinary angiostatin were associated with the more severity of pathological lesions. In the study, we also found that urinary angiostatin levels were positively associated with 24-h urinary protein excretion, serum creatinine and cystatin C and negatively associated with eGFR, all of which were well-established predictive markers for IgAN disease progression. Proteinuria may have consistent and independent associations with a worse prognosis [10]. These findings suggest that urinary angiostatin was closely associated with kidney damage. Patients with more severe kidney damage may exhibit higher levels of urinary angiostatin.

However, the elevated level of urinary angiostatin did not simply occur in patients with advanced IgAN. A previous study [11] found that urinary angiostatin levels were significantly higher in patients with systemic lupus erythematosus (LN) than in healthy controls and other CKD patients. Patients with class IV LN exhibited the highest levels. They also found urinary angiostatin levels were well correlated with the renal pathological chronicity index and may reflect the prognosis of LN patients. In our study, we also found that urinary angiostatin expression was low in healthy controls subjects without renal injury. Furthermore, the patients with higher urinary angiostatin levels easily reached composite outcome and had a worse prognosis. These findings suggest that urinary angiostatin can not only be used as a specific biomarker for IgAN or LN, it may also be a marker of renal disease severity and reflect the degree of disease progression.

Angiostatin, a 38-kDa plasminogen fragment that was discovered in the serum and urine of tumor-bearing mice, has anti-angiogenic and anticancer effects [12]. Unequivocal evidence suggests that angiogenesis-related factors (e.g., vascular endothelial growth factor, angiopoietins, thrombospondin-1, angiostatin, and endostatin) play a pathogenic role in the advanced stages of CKD [13]. A recent study found that angiostatin was elevated in patients with CKD and associated with an improvement in chronic kidney injury in the rat remnant kidney model [4].

Angiostatin is a potent angiogenic inhibitor that blocks proliferation, induces apoptosis, prevents migration of endothelial cells, and disrupts capillary integrity [12]. Studies found that elevated angiostatin expression in the kidney was related to renal capillary density loss and interstitial damage [14, 15], which resulted in loss of glomerular function. Our study has demonstrated that angiostatin levels were higher in patients with severe tubule-interstitial lesions, and the significant correlation occurred between the biomarker and 24-h urinary protein excretion. The elevated level of angiostatin expression may aggravate tubule-interstitial damage and proteinuria. In addition, angiostatin has anti-inflammatory actions by inhibiting leukocyte recruitment and both neutrophil and macrophage migration [16].

Studies have shown that angiogenic factors inhibit the progression of CKD and are recognized as novel therapeutic approaches to protect the kidney [13]. The role of angiogenic factors in kidney disease drew attention because of the effect on the balance between renal angiogenesis and inflammation. Studies that attempted to treat kidney disease with angiostatin found that angiostatin might have a therapeutic effect on kidney disease in vivo. They found that angiostatin had an anti-inflammatory effect, reduced proteinuria, and ultimately played a role in improving kidney inflammation and interstitial fibrosis in the rat remnant kidney model [4]. Similarly, treatment with recombinant adeno-associated viruses expressing angiostatin could ameliorate albuminuria and glomerular hypertrophy in diabetic rats [17]. However, inflammatory factors and angiostatin generation were both increased in L-NAME-treated aged rat kidney, accompanied by increased cathepsin D activation [15]. Anti-angiogenic actions of angiostatin offset its benefits in CKD. And, its inflammatory effect is worth investigating. At present, the exact role of angiostatin in CKD patients remains unclear due to the lack of direct evidence of angiostatin acting on kidney damage.

In this study, we did not measure expression of serum and kidney angiostatin in IgAN patients and control subjects. The major source of the increased urine angiostatin level remains unknown [17]. However, the kidney, particularly the tubular cells but not serum, was thought to produce or enrich angiostatin locally in patients with LN [11]. The production of angiostatin in the urine may be the activation of plasminogen by human urinary urokinase via Cl^−^-binding modulation. Renal angiostatin production was also thought to be related to a possible role for matrix metalloproteinase (MMPs) and decreased cathepsin D activity [14, 15]. Accompanied with MMP-2 and MMP-9 enhancement in post-ischemic kidney tissue and localization to the renal tubules, both renal and urinary angiostatin levels were dramatically elevated in rats with ischemic renal injury. Cathepsin D is also responsible for angiostatin generation [18] in human prostate carcinoma cells. Its activity was increased, accompanied by an increase in angiostatin production in the aging rat kidney and vice versa. Renal plasminogen activation and filtration may be the major source of urinary angiostatin.

## Conclusions

In summary, IgAN patients with severe clinical and pathological lesions present with higher urinary angiostatin levels. Urinary angiostatin may reflect IgAN disease severity, predict prognosis, and can be used as a noninvasive biomarker of severe IgAN.

## Additional file

Additional file 1: **Table S1.** Demographics and clinical characteristics of patients in the IgAN group. **Table S2.** Clinical characteristics of healthy subjects. **Table S3.** Lee’s grade and Oxford Classification scores characteristics of patients in the IgAN group. **Table S4.** Demographics and clinical characteristics of patients in the matched IgAN group and DC controls. (DOCX 16 kb)

## Abbreviations

angiostatin/Cr: the concentration of angiostatin in urine normalized to urinary creatinine
ANOVA: One-way analysis of variance
CKD: Chronic kidney disease
DC: disease controls
E: Endocapillary hypercellularity
eGFR: Estimated glomerular filtration rates
ELISA: Enzyme-linked immunosorbent assay
ESRD: End-stage renal disease
FSGS: Focal segmental glomerulosclerosis
HC: Healthy controls
IgAN: IgA Nephropathy
IQR: Interquartile range
LN: Systemic lupus erythematosus
M: mesangial proliferative score
MCD: Minimal change nephropathy
MMPs: Matrix metalloproteinase
MN: membranous nephropathy
S: Segmental glomerulosclerosis
SD: Standard deviation
T: Tubular atrophy/interstitial fibrosis score

## References

[1] Li LS, Liu ZH (2004). Epidemiologic data of renal diseases from a single unit in China: analysis based on 13,519 renal biopsies. Kidney Int.

[2] Donadio JV, Grande JP (2002). IgA nephropathy. N Engl J Med.

[3] D'Amico G (2000). Natural history of idiopathic IgA nephropathy: role of clinical and histological prognostic factors. Am J Kidney Dis.

[4] Cattran DC, Coppo R, Cook HT, Feehally J, Roberts IS, Troyanov S, Alpers CE, Amore A, Barratt J, Berthoux F (2009). The Oxford classification of IgA nephropathy: rationale, clinicopathological correlations, and classification. Kidney Int.

[5] Lv J, Shi S, Xu D, Zhang H, Troyanov S, Cattran DC, Wang H (2013). Evaluation of the Oxford classification of IgA nephropathy: a systematic review and meta-analysis. Am J Kidney Dis.

[6] Zhao N, Hou P, Lv J, Moldoveanu Z, Li Y, Kiryluk K, Gharavi AG, Novak J, Zhang H (2012). The level of galactose-deficient IgA1 in the sera of patients with IgA nephropathy is associated with disease progression. Kidney Int.

[7] Rocchetti MT, Papale M, d'Apollo AM, Suriano IV, Di Palma AM, Vocino G, Montemurno E, Varraso L, Grandaliano G, Di Paolo S (2013). Association of urinary laminin G-like 3 and free K light chains with disease activity and histological injury in IgA nephropathy. Clin J Am Soc Nephrol.

[8] Zhao Y, Zhu L, Zhou T, Zhang Q, Shi S, Liu L, Lv J, Zhang H (2015). Urinary CXCL1: a novel predictor of IgA nephropathy progression. PLoS One.

[9] von Eggeling F, Davies H, Lomas L, Fiedler W, Junker K, Claussen U, Ernst G (2000). Tissue-specific microdissection coupled with ProteinChip array technologies: applications in cancer research. BioTechniques.

[10] Barbour SJ, Reich HN (2012). Risk stratification of patients with IgA nephropathy. Am J Kidney Dis.

[11] Wu T, Du Y, Han J, Singh S, Xie C, Guo Y, Zhou XJ, Ahn C, Saxena R, Mohan C (2013). Urinary angiostatin--a novel putative marker of renal pathology chronicity in lupus nephritis. Mol Cell Proteomics.

[12] O'Reilly MS, Holmgren L, Shing Y, Chen C, Rosenthal RA, Moses M, Lane WS, Cao Y, Sage EH, Folkman J (1994). Angiostatin: a novel angiogenesis inhibitor that mediates the suppression of metastases by a Lewis lung carcinoma. Cell.

[13] Tanaka T, Nangaku M (2013). Angiogenesis and hypoxia in the kidney. Nat Rev Nephrol.

[14] Basile DP, Fredrich K, Weihrauch D, Hattan N, Chilian WM (2004). Angiostatin and matrix metalloprotease expression following ischemic acute renal failure. Am J Physiol Renal Physiol.

[15] Satoh M, Kidokoro K, Ozeki M, Nagasu H, Nishi Y, Ihoriya C, Fujimoto S, Sasaki T, Kashihara N (2013). Angiostatin production increases in response to decreased nitric oxide in aging rat kidney. Lab Invest.

[16] Aulakh GK, Balachandran Y, Liu L, Singh B (2014). Angiostatin inhibits activation and migration of neutrophils. Cell Tissue Res.

[17] Zhang SX, Wang JJ, Lu K, Mott R, Longeras R, Ma JX (2006). Therapeutic potential of angiostatin in diabetic nephropathy. J Am Soc Nephrol.

[18] Morikawa W, Yamamoto K, Ishikawa S, Takemoto S, Ono M, Fukushi J, Naito S, Nozaki C, Iwanaga S, Kuwano M (2000). Angiostatin generation by cathepsin D secreted by human prostate carcinoma cells. J Biol Chem.

