# Supplementary material for: Urinary angiostatin: a novel biomarker of kidney disease associated with disease severity and progression
Source: BMC Nephrol. 2019 Apr 3;20:118. doi: 10.1186/s12882-019-1305-2 (PMC6446319; doi:10.1186/s12882-019-1305-2)
Supplement: Supplementary file 1 — Table S1. Demographics and clinical characteristics of patients in the IgAN group. Table S2. Clinical characteristics of healthy subjects. Table S3. Lee’s grade and Oxford Classification scores characteristics of patients in the IgAN group. Table S4. Demographics and clinical characteristics of patients in the matched IgAN group and DC controls. (DOCX 16 kb) [file 12882_2019_1305_MOESM1_ESM.docx]

**Table1S.** Demographics and clinical characteristics of patients in the IgAN group.

|  | IgAN |
| --- | --- |
| Sex(male/female) | 12/3 |
| Age(years) | 29.33±8.73 |
| SBP(mmHg) | 126.35±18.43 |
| DBP(mmHg) | 80.59±13.64 |
| Scr(μmol/L） | 128.6±82.9 |
| Proteinuria（g/24h) | 1.71±1.05 |
| eGFR(ml/ min per 1.73 m²) | 87.05±37.76 |

**Table2S.** Clinical characteristics of healthy subjects.

|  | HC |
| --- | --- |
| Sex(male/female) | 24/16 |
| Age(years) | 43.78±14.37 |
| MAP(mmHg) | 84.91±2.67 |
| TP (g/L) | 70.04±4.78 |
| Alb (g/L) | 45.52±3.55 |
| Scr (μmol/L) | 65.25±5.20 |
| UA (μmol/L) | 295.4±53.51 |

**Table3S.** Lee’s grade and Oxford Classification scores characteristics of patients in the IgAN group.

|  | IgAN |
| --- | --- |
| Lee’s grade I-II | 15 |
| Lee’s grade III | 90 |
| Lee’s grade IV-V | 72 |
| M0/M1 | 78/99 |
| E0/E1 | 153/24 |
| S0/S1 | 60/117 |
| T0/T1/T2 | 76/43/58 |

**Table4S.** Demographics and clinical characteristics of patients in the matched IgAN group and DC controls.

|  | IgAN | DC | P value |
| --- | --- | --- | --- |
| Sex(male/female) | 49/36 | 48/37 | 0.855 |
| Age(years) | 33.58±12.5 | 44.44±13.19 | <0.001 |
| MAP(mmHg) | 99.97±12.63 | 98.35±11.36 | 0.382 |
| TP (g/L) | 64.95±9.63 | 54.41±11.48 | <0.001 |
| Alb (g/L) | 37.29±7.37 | 30.06±8.91 | <0.001 |
| Scr (μmol/L) | 92.80(71.35, 125.3) | 85.20(60.85, 117.35) | 0.154 |
| UA (μmol/L) | 378.85±99.82 | 363.74±109.16 | 0.348 |
| BUN (mmol/L) | 5.45±2.01 | 7.13±5.43 | 0.008 |
| Proteinuria (g/24h)  <1 n(%)  1～3.5 n(%)  ≥3.5 n(%) | 1.56(0.83,3.57)  25(29.4)  39(45.9)  21(24.7) | 2.05(0.90,4.20)  24(28.2)  36(42.4)  25(29.4) | 0.225  0.612 |
| eGFR (ml/min per 1.73m²) | 81.09±32.96 | 84.21±30.04 | 0.519 |
| CKD stage  CKD1 stage n(%)  CKD2 stage n(%)  CKD3 stage n(%)  CKD4 stage n(%) | 85  38（44.7）  20（23.5）  22（25.9）  5(5.9) | 85  45(52.9)  20(23.5)  15（17.6）  5（5.9） | 0.240 |
